# Predicting longitudinal brain atrophy in Parkinson’s disease using a Susceptible-Infected-Removed agent-based model

**DOI:** 10.1162/netn_a_00296

**Published:** 2023-10-01

**Authors:** Alaa Abdelgawad, Shady Rahayel, Ying-Qiu Zheng, Christina Tremblay, Andrew Vo, Bratislav Misic, Alain Dagher

**Affiliations:** The Neuro (Montreal Neurological Institute-Hospital), McGill University, Montreal, Canada; Centre for Advanced Research in Sleep Medicine, Hôpital du Sacré-Cœur de Montréal, Montreal, Canada; Wellcome Centre for Integrative Neuroimaging, Centre for Functional Magnetic Resonance Imaging of the Brain, University of Oxford, John Radcliffe Hospital, Oxford, United Kingdom

**Keywords:** MRI, Alpha-synuclein, Brain networks, Neurodegeneration

## Abstract

Parkinson’s disease is a progressive neurodegenerative disorder characterized by accumulation of abnormal isoforms of alpha-synuclein. Alpha-synuclein is proposed to act as a prion in Parkinson’s disease: In its misfolded pathologic state, it favors the misfolding of normal alpha-synuclein molecules, spreads trans-neuronally, and causes neuronal damage as it accumulates. This theory remains controversial. We have previously developed a Susceptible-Infected-Removed (SIR) computational model that simulates the templating, propagation, and toxicity of alpha-synuclein molecules in the brain. In this study, we test this model with longitudinal MRI collected over 4 years from the Parkinson’s Progression Markers Initiative (1,068 T1 MRI scans, 790 Parkinson’s disease scans, and 278 matched control scans). We find that brain deformation progresses in subcortical and cortical regions. The SIR model recapitulates the spatiotemporal distribution of brain atrophy observed in Parkinson’s disease. We show that connectome topology and geometry significantly contribute to model fit. We also show that the spatial expression of two genes implicated in alpha-synuclein synthesis and clearance, *SNCA* and *GBA*, also influences the atrophy pattern. We conclude that the progression of atrophy in Parkinson’s disease is consistent with the prion-like hypothesis and that the SIR model is a promising tool to investigate multifactorial neurodegenerative diseases over time.

## Author Summary

It has been suggested that neurodegenerative diseases are caused by abnormal generation, propagation, and accumulation of neurotoxic protein isoforms. In the case of Parkinson’s disease, the culprit is alpha-synuclein, which has been shown in animal models to exhibit prion-like properties. We devised a Susceptible-Infected-Removed agent-based model of synucleinopathy, in which we simulate the fates of normal and misfolded alpha-synuclein molecules moving along a brain network. We show that the progression of brain atrophy in Parkinson’s disease over the first 2 years following diagnosis can be simulated by our model. We conclude that the pattern of brain atrophy in Parkinson’s disease is shaped by a combination of the connectome and regional expression of genes that control the clearance and synthesis of alpha-synuclein. Our work supports the prion-like model of neurodegeneration in Parkinson’s disease.

## INTRODUCTION

Parkinson’s disease (PD) is characterized by the pathological intracellular aggregation of misfolded alpha-synuclein (aSyn) into Lewy bodies and neurites (Dickson et al., 2009; Spillantini et al., 1997). In the brain, these deposits often appear in a stereotypical fashion, emerging in the olfactory bulb and caudal brainstem and then ascending towards the midbrain, limbic areas, and cerebral cortex (Braak et al., 2003; Braak, Ghebremedhin, Rüb, Bratzke, & Del Tredici, 2004). This spatiotemporal distribution pattern of pathology has led to the hypothesis that misfolded aSyn may harbor prion-like properties (Brundin & Melki, 2017), allowing it to spread between cells and impose its misfolded conformation onto native endogenous, otherwise normal aSyn from the recipient cell (Peng, Trojanowski, & Lee, 2020). Indeed, the injection of synthetic aSyn preformed fibrils or brain lysates from patients with a synucleinopathy has demonstrated the local formation of aSyn pathology and its propagation through brain networks in wild-type and transgenic mice, rats, and nonhuman primates (Henrich et al., 2020; Luk et al., 2012; Masuda-Suzukake et al., 2013; Rey et al., 2018; Rey et al., 2016; Uemura, Uemura, Luk, Lee, & Trojanowski, 2020; Watts et al., 2013).

In humans, the evidence for a prion-like behavior of pathological aSyn has so far been indirect. For instance, in patients who received fetal mesencephalic neuronal transplants, Lewy-related pathology could be observed inside cells that were grafted a decade earlier (Kordower, Chu, Hauser, Freeman, & Olanow, 2008; Li et al., 2008), suggesting that pathology spread to the grafts from the surrounding milieu. Also, using MRI-derived volume deformation and cortical thinning as proxy measures of tissue atrophy, the pattern of brain changes observed in de novo PD patients was shown to significantly overlap with the brain’s connectivity pattern (Pandya et al., 2019; Tremblay et al., 2021; Zeighami et al., 2015). However, other studies have also shown that the distribution of aSyn pathology is not solely explainable by brain connectivity and that other cell-autonomous factors play a role in shaping aSyn pathology (Gonzalez-Rodriguez, Zampese, & Surmeier, 2020; Henrich et al., 2020; Surmeier, Obeso, & Halliday, 2017). Thus, the mechanisms underlying the accumulation and propagation of pathological aSyn in PD remain unclear.

One way to understand these mechanisms is through computational modeling. We have recently developed an agent-based Susceptible-Infected-Removed (SIR) model that simulates the fate of individual aSyn proteins in the brain to recreate, based on cell-autonomous factors and brain connectivity, the atrophy pattern seen in PD (Zheng et al., 2019). The local factors in this case are expression of the genes *SNCA* and *GBA*, which we take as proxies of synthesis and clearance of aSyn. Using this model, we previously recreated the pattern of atrophy observed at baseline in de novo PD patients from the Parkinson’s Progression Markers Initiative (PPMI) cohort and demonstrated that both structural connectivity and gene expression contributed to model accuracy (Zheng et al., 2019). We subsequently used the same model to demonstrate that gene and connectivity factors also underpinned the propagation of pathologic aSyn injected into different brain regions of wild-type mice (Rahayel, Misic, et al., 2022). Moreover, we also demonstrated that the brain atrophy seen in patients with isolated REM sleep behavior disorder, a prodromal state of PD and dementia with Lewy bodies (Postuma et al., 2019), could also be recreated with the model (Rahayel, Tremblay, et al., 2022). However, it remains unknown whether the propagation of aSyn in the brain also shapes the progression of brain atrophy in PD over several years.

In this study, our objective was to investigate whether the agent-based SIR model could recreate the longitudinal patterns of brain atrophy in PD patients from the PPMI cohort over 4 years. We used deformation-based morphometry to measure the progression of tissue deformation (atrophy) in PD patients versus controls over 1, 2, and 4 years. We then applied the agent-based SIR model to assess whether *SNCA* and *GBA* gene expression and structural features of the connectome significantly contributed to recreating the atrophy patterns. We found that the agent-based SIR model recreated the atrophy observed longitudinally in PD and that both gene expression and connectivity are significant contributors to the distribution of atrophy.

## METHODS

### Participants

Longitudinal data from 790 PD patients and 278 healthy control participants were included from the PPMI database (https://www.ppmi-info.org), for a total of 1,068 MRI scans and associated clinical measures. The PPMI is a longitudinal observational international study aimed at assessing progression markers of PD and includes a comprehensive set of clinical and MRI measures acquired in patients with de novo PD and in healthy controls (Marek et al., 2018).

To be included in the PPMI, PD patients (a) had at least two features among resting tremor, bradykinesia, and rigidity or either asymmetric resting tremor or asymmetric bradykinesia; (b) had a diagnosis of PD for less than 2 years; (c) had a baseline Hoehn and Yahr stage of I or II; (d) had a dopamine transporter binding deficit confirmed using single photon emission computed tomography; (e) were not expected to require medications for PD within six months of the baseline assessment; (f) were at least 30 years old; and (g) did not have dementia. For healthy controls, a Montreal Cognitive Assessment score below 27 or a first-degree relative with a clinical diagnosis of idiopathic PD led to exclusion. The longitudinal follow-up of PPMI now extends to approximately 5 years; for this study, only the participants with MRI acquisition performed at baseline and at either 1, 2, and/or 4 years were considered for analysis because of the limited number of scans acquired at 3 years (three participants) and 5 years (two participants). The PPMI investigators obtained informed consent from all participants. This research using derived data was approved by the Research Ethics Board of the McGill University Health Center.

### Clinical Measures

At each visit, patients underwent the Movement Disorders Society-Unified Parkinson’s Disease Rating Scale (MDS-UPDRS; Goetz et al., 2007), the Montreal Cognitive Assessment (Nasreddine et al., 2005), the REM Sleep Behavior Disorder Screening Questionnaire, on which a score ≥5 indicates probable REM sleep behavior disorder (Stiasny-Kolster et al., 2007), the Geriatric Depression Scale, the State-Trait Anxiety Inventory, and the Scales for Outcomes in Parkinson’s Disease-Autonomic.

### Cognitive Assessment

In addition to the aforementioned evaluation, every patient also underwent a comprehensive cognitive assessment that included the Symbol-Digit Modalities Test; the Letter-Number Sequencing test; the Benton Judgment of Line Orientation test; the semantic and phonemic fluency tasks; and the total recall, delayed recall, and recognition tasks from the Hopkins Verbal Learning Test-Revised (Weintraub et al., 2015).

### MRI Acquisition

T1-weighted MRI brain images were acquired at different sites across the United States, Canada, and Europe, with the following parameters: repetition time = 2,300 ms; echo time = 2.98 ms; field of view = 256 mm; flip angle = 9; and voxel size = 1 mm^3^. The acquisition protocols are available on the PPMI website (https://www.ppmi-info.org/study-design/research-documents-and-sops/).

### Deformation-Based Morphometry

Deformation-based morphometry (DBM) was performed on the baseline and longitudinal T1-weighted scans of PD patients and controls to derive whole-brain individual atrophy maps. Specifically, the maps represent the deformation needed for each voxel to be normalized to the template space. DBM was done using the default parameters available in the CAT12 toolbox in SPM12 (https://www.neuro.uni-jena.de/cat). This resulted in a set of processed image files for each participant that included a voxel-wise whole-brain map of Jacobian determinants, which was used as the measure of local brain tissue atrophy after the application of a 2-mm full width at half maximum isotropic smoothing kernel. Images were visually inspected at each step and excluded if abnormal or if the quality rating score was below 80%. The image quality rating score is an aggregate measurement generated by the CAT12 processing pipeline; it integrates several metrics of image quality, namely noise-to-contrast ratio, coefficient of joint variation, inhomogeneity-to-contrast ratio, and root-mean-squared voxel resolution into a single value ranging from 0 to 1 (i.e., the higher the score, the better the image quality).

### Brain Parcellation

The normalized smoothed Jacobian determinants maps were next parcellated using a previously used atlas made of 42 cortical and subcortical brain regions from the left hemisphere for which regional *SNCA* and *GBA* expression as well as structural connectome features were available (Zheng et al., 2019). This atlas included 34 cortical regions derived from the Desikan-Killiany Atlas (Desikan et al., 2006) and 7 subcortical regions, namely the putamen, caudate, pallidum, thalamus, hippocampus, amygdala, and accumbens, available as part of the FreeSurfer processing stream (https://surfer.nmr.mgh.harvard.edu). Because of its importance in PD, the substantia nigra was additionally included based on the segmentation available from a 7T MRI basal ganglia atlas (https://www.nitrc.org/projects/atag; Keuken et al., 2014). Using FLIRT (https://fsl.fmrib.ox.ac.uk/fsl/fslwiki/FLIRT), the 42-region atlas was then linearly registered to the individual deformation maps and a set of 42 regional deformation values were extracted for each map using the MarsBaR region of interest toolbox for SPM (https://marsbar.sourceforge.net). Note that the atlas only included regions from the left hemisphere due to the *SNCA* and *GBA* gene expression for the right hemisphere being available for only two of the six postmortem brains in the Allen Human Brain Atlas (Hawrylycz et al., 2012). We also tested the model on one hemisphere to avoid possible errors associated with the detection of interhemispheric white matter connections with deterministic streamline tractography (see below).

### Regional Atrophy Quantification

**Figure 1.**
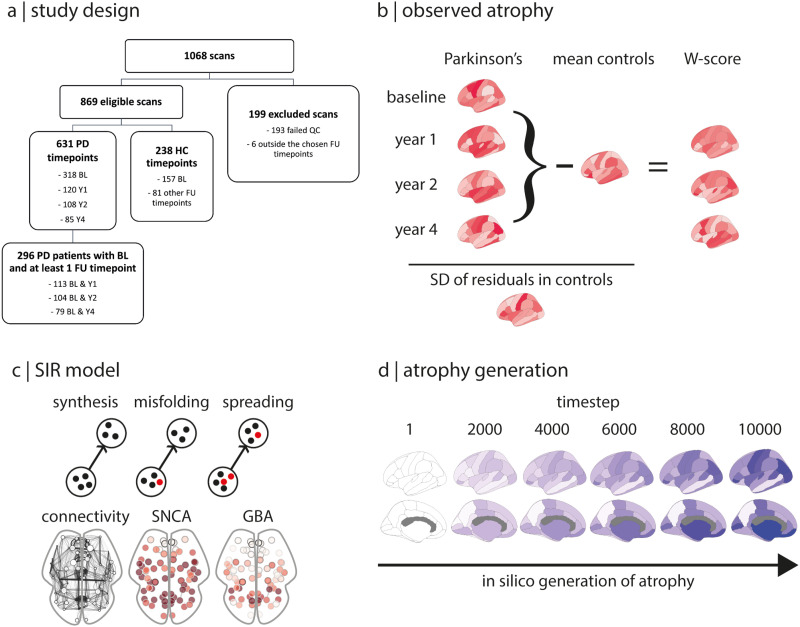
Overview. (A) Study design. (B) Deformation-based morphometry was performed on each scan. W-scored parcellated maps were generated for each follow-up timepoint by correcting the atrophy maps for the effects of age and sex seen in controls. The baseline W-scored map was subtracted from each of the follow-up W-scored maps to generate progression maps of atrophy at 1, 2, and 4 years, yielding three maps of brain atrophy progression in PD. The spread of alpha-synuclein pathology in the brain was simulated with the agent-based SIR model based on structural connectivity and gene expression of *SNCA* and *GBA*. (D) The propagation of agents and the resulting atrophy was simulated over 10,000 timesteps, with a brain atrophy map generated at each timestep. The observed atrophy maps were compared with the simulated brain atrophy maps. BL = baseline; FU = follow-up; HC = healthy controls; PD = Parkinson’s disease; QC = quality control; SD = standard deviation; SIR = Susceptible-Infected-Removed.

A W-score approach was used to account for the normal effects of age and sex on brain morphometry (La Joie et al., 2012; Tremblay et al., 2020). The regional deformation values from each PD patient’s MRIs were converted into age- and sex-corrected W-scores based on the values observed in the 157 controls available at baseline. There was no significant difference in age and sex between the controls (age: 60.1 ± 11.9, 66% male) at baseline and the PD group at each follow-up timepoint (baseline age: 60.9 ± 10.0, 63% male; year 1 age: 60.9 ± 9.3, 63% male; year 2 age: 60.9 ± 9.3, 63% male; year 4 age: 64.4 ± 9.9, 69% male). Only the values from controls seen at baseline were used for standardization because of the limited number of controls who underwent follow-up MRI. The W-score formula was the following:

$$\text{Wscore}=\frac{{PD}_{\text{raw}\;\text{value}}-{PD}_{\text{predicted}\;\text{based}\;\text{on}\;\text{HC}}}{{SD}_{\text{residuals}\;\text{in}\;\text{HC}}},$$

where the predicted value for a PD patient based on control data was given by (*β*_1_ × age + *β*_2_ × sex + *β*_3_). In other words, this yielded regional deformation values that represented the difference between a PD patient’s deformation value and the deformation value that is expected for their age and sex. Here, W-scores are essentially z-scores corrected for age and sex (La Joie et al., 2012). In this work, only age and sex were used as control variables, whereas all other clinical and cognitive measures were exclusively used for characterizing the PD group at baseline and over time. The individual W-scores were then averaged between patients for a given region, resulting in a set of 42 regional W-scores for baseline and each of the three follow-up timepoints. The baseline W-score was then subtracted from the average W-score seen at each follow-up timepoint to yield a W-score difference over time (i.e., atrophy progression over 1, 2, and 4 years). These were inverted for interpretability: A positive W-score indicated atrophy progression, whereas a negative W-score difference represented volume expansion. The three sets of 42 atrophy difference values, one for the difference between baseline and every follow-up timepoint, were the observed patterns of atrophy progression to which we compared the pattern of simulated atrophy generated in silico by the agent-based SIR model. An overview of the analytical steps is presented in Figure 1.

### Agent-Based SIR Model

#### Overview of the model.

The agent-based SIR model is a computational model based on infectious disease epidemiology; however, instead of studying the propagation of an infection in a population, it simulates the propagation of aSyn in a brain network. In our model, the agents are aSyn molecules, which can belong to one of three compartments: “susceptible” when representing the normal protein, “infected” when representing the misfolded protein, and “removed” when representing a protein that is no longer active in the region. This model has been shown to accurately recreate the distribution of pathology in non-transgenic mice injected with preformed aSyn fibrils as well as the atrophy pattern observed in patients with PD (at baseline), isolated REM sleep behavior disorder, and frontotemporal dementia (Rahayel, Misic, et al., 2022; Rahayel, Tremblay, et al., 2022; Shafiei et al., 2021; Zheng et al., 2019).

This model of disease spread starts by injecting pathology in one seed region and allowing it to propagate through brain networks constrained by regional characteristics such as gene expression and connectivity. When applied to aSyn, this model uses the regional expression of the *SNCA* and *GBA* genes as indices of the local synthesis and degradation of aSyn agents, respectively, and the connectivity strength between brain regions based on diffusion MRI to modulate the spread of agents between regions. The transition between compartments is determined by a set of rules that guide the interactions between agents and their local environment. All Infected agents are deemed to be infectious, and every susceptible agent can turn into an Infected agent when it encounters an infected agent. Susceptible and infected agents can travel between brain regions to spread pathology. Both susceptible and infected agents in a region have a probability of turning into removed agents, either by being degraded inside the region or by spreading to a connected area.

In this study, pathology was initiated in the substantia nigra and its propagation through the brain was simulated over a total of 10,000 iterations. At each iteration, an atrophy value was calculated in every brain region, derived from the combined effects of the local accumulation of infected agents and the ongoing deafferentation between brain regions (cell death). To assess whether the SIR model and its gene expression and connectivity parameters significantly recreated atrophy progression in PD, the atrophy resulting from aSyn spread was simulated in silico for the 42 brain regions and compared with the patterns of atrophy progression observed between baseline and 1, 2, and 4 years in PD patients. In other words, following the initiation of pathology in the substantia nigra, the model used information about structural connectivity and regional gene expression to modulate the behavior of aSyn agents and simulate the local accumulation of aSyn pathology and atrophy.

The model was implemented as five different modules (production of normal aSyn, clearance of normal and misfolded aSyn, misfolding of normal aSyn, propagation of normal and misfolded aSyn, accrual of atrophy), which are described in the following sections.

#### Production of normal aSyn.

In the model, the synthesis of aSyn inside every region was modulated based on the regional gene expression of *SNCA*, which was extracted using *abagen* (Markello et al., 2021), available at https://abagen.readthedocs.io/, for the 42 regions based on the six postmortem brains from the Allen Human Brain Atlas (Hawrylycz et al., 2012). The values were averaged across samples to yield an expression vector of synthesis that was inserted back into the model (Zheng et al., 2019). The synthesis rate in region *i* is given as the probability of new agent synthesis per unit time, *α*_*i*_:

$$\alpha_{i}=\Phi_{0,1}\left(\text{SNCA}{\text{expression}}_{i}\right),$$

where Φ_0,1_(·) is the normal cumulative distribution function. The increment of normal agents in region *i* is given by *α*_*i*_S_*i*_Δ*t*, where Δ*t* is the total time and S_*i*_ is the region size. The time increment used for the main analyses was set at Δ*t* = 0.1, but peak correlation fits were robust with values from 0.1 to 0.9 (see Supplementary Figure 1 in the Supporting Information).

#### Clearance of normal and misfolded aSyn.

Similarly, the degradation of aSyn inside each region was dependent on the regional gene expression of *GBA*, which was also extracted from the Allen Human Brain Atlas. The clearance rate of both normal and misfolded agents in region *i* per unit time occurred with the probability distribution *β*_*i*_:

$$\beta_{i}=\Phi_{0,1}\left(\text{GBA}{\text{expression}}_{i}\right),$$

where Φ_0,1_(·) is the normal cumulative distribution function. The probability of an agent still being active after total time Δ*t* is given by lim_*δτ*→0_(1 − *βδτ*)^Δ*t*/*δτ*^ = *e*^−*β*Δ*t*^. In other words, as the degradation rate increases, the probability of an agent to remain active in the region decreases. Accordingly, the proportion of cleared agents within timestep Δ*t* is 1 − *e*^−*β*Δ*t*^.

#### Misfolding of normal aSyn (infection transmission).

Infected agents can promote misfolding of susceptible agents and turn them into infected agents. The probability of a susceptible agent that survived clearance of not becoming infected is (1 − $\gamma_{i}^{0}$)^*M*_*i*_^, where *M*_*i*_ is the population of infected agents in region *i* and $\gamma_{i}^{0}$ is the baseline likelihood that a single misfolded agent turns a susceptible agent into an infected agent. The baseline likelihood $\gamma_{i}^{0}$ is given by 1/*S*_*i*_, where *S*_*i*_ is the region size. Accordingly, the probability per unit time that a susceptible agent surviving clearance in region *i* turns into an infected agent due to the action of at least one of the *M*_*i*_ infected agents present in region *i* is given by *γ*_*i*_ = 1 − *e*^*M*_*i*_ ln(1−$\gamma_{i}^{0}$)^. As for the previous module, the probability that a susceptible agent remains susceptible after total time Δ*t* is given by lim_*δx03C4;*→0_(1 − $\gamma_{i}^{0}$*δτ*)^*M*_*i*_Δ*t*/*δτ*^ = *e*^−$\gamma_{i}^{0}$*M*_*i*_Δ*t*^, whereas the probability that a susceptible agent becomes infected after total time Δ*t* is given by 1 − *e*^−$\gamma_{i}^{0}$*M*_*i*_Δ*t*^. As a result, the increment of the population of normal proteins *N*_*i*_ in region *i* is the following:

$$\Delta N_{i}=\alpha_{i}S_{i}\Delta t-\left(1-e^{-\beta_{i}\Delta t}\right)N_{i}.$$

After each timestep, the populations of susceptible (*N*_*i*_) and infected agents (*M*_*i*_) are respectively updated as follows:

$$\Delta N_{i}=\alpha_{i}S_{i}\Delta t-\left(1-e^{-\beta_{i}\Delta t}\right)N_{i}-\left(e^{-\beta_{i}\Delta t}\right)\left(1-e^{-\gamma_{i}^{0}M_{i}\Delta t}\right)N_{i},$$

$$\Delta M_{i}=\left(e^{-\beta_{i}\Delta t}\right)\left(1-e^{-\gamma_{i}^{0}M_{i}\Delta t}\right)N_{i}-\left(1-e^{-\beta_{i}\Delta t}\right)M_{i}.$$

#### Propagation of normal and misfolded aSyn.

Every susceptible and infected agent has a probability to spread to other brain regions via fiber tracts (edges in the network). To implement this in the model, we used a previously created structural connectivity matrix of the complete interregional connection strength values for the same 42 regions used for quantifying atrophy (Zheng et al., 2019). The structural connectivity matrix was created from the preprocessed diffusion-weighted and T1-weighted MRI images from 1,027 healthy participants from the Human Connectome Project (2017 Q4; 1,200-subject release). The diffusion data were reconstructed in the individual T1 spaces using generalized q-sampling imaging with a diffusion sampling ratio of 1.0 to generate voxel-wise maps of quantitative anisotropy and spin distribution function. Deterministic streamline tractography was then performed in native space using DSI Studio (https://www.nitrc.org/projects/dsistudio) with an angular cutoff of 55, a step size of 0.5 mm, a minimum length of 20 mm, and a maximum length of 400 mm to reconstruct 100,000 streamlines for every region. Group-level matrices of connectivity strength and distance were then generated. The connectivity matrix represents the density of streamlines (streamline counts) between each region pair normalized by the target region volume (voxel counts). This connectivity strength was measured with every region entered as the tractography seed region to generate a whole-brain structural connectivity profile that covers the 42 regions used for atrophy measurement. For each subject, the connectivity profiles were concatenated to form a 42 × 42 matrix where every element represents the connection strength between every pair of regions, with self-connections set to 0. A group-consensus approach was then performed by averaging the connectivity profiles and keeping 35% of the most commonly occurring edges across all subjects. This group-level matrix of structural connectivity was used in the SIR model for computing the probability of an agent spreading between regions (see below). To ensure that findings were not due to the density threshold of retained connections, we repeated the analyses using different matrix densities (Supplementary Table 1 in the Supporting Information). In addition to this connection strength matrix, we also generated a group-level connectivity distance matrix based on the mean Euclidean distance of corresponding streamlines between every pair of regions. This distance matrix was also used to modulate the mobility pattern of agents between regions as explained below.

At each timestep, every agent can either remain in region *i* or enter the edges (i.e., fiber tracts connecting two regions) with probabilities:

$$P_{{\text{region}}_{i}\to {\text{region}}_{i}}=\rho_{i},$$

$$P_{{\text{region}}_{i}\to {\text{edge}}_{ij}}=\left(1-\rho_{i}\right)\frac{w_{ij}}{\sum_{j}w_{ij}},$$

where *w*_*ij*_ is the undirected connection strength between region *i* and region *j* and *ρ*_*i*_ is the probability of an agent to remain in region *i*. This probability was set to 0.5 for every region. The choice of *ρ*_*i*_ led to negligible differences when simulating atrophy (Supplementary Figure 1 in the Supporting Information). Similarly, for susceptible and infected agents in an edge, the probability of leaving the edge to enter another region per unit time is given by these probabilities:

$$P_{{\text{edge}}_{ij}\to {\text{region}}_{j}}=\frac{1}{l_{ij}},$$

$$P_{{\text{edge}}_{ij}\to {\text{edge}}_{ij}}=1-\frac{1}{l_{ij}},$$

where *l*_*ij*_ is the length (Euclidean distance) of the edge between regions *i* and *j*. The increments in quantity of normal and misfolded agents *N*_*i*_ and *M*_*i*_ in region *i* after a total time Δ*t* are as follows:

$$\Delta N_{i}=\sum_{j}\frac{1}{l_{ji}}N_{ji}\Delta t-\left(1-\rho_{i}\right)N_{i}\Delta t,$$

$$\Delta M_{i}=\sum_{j}\frac{1}{l_{ji}}M_{ji}\Delta t-\left(1-\rho_{i}\right)M_{i}\Delta t,$$

where *N*_*ij*_ and *M*_*ij*_ represent the populations of normal and infected agents in the edge between regions *i* and *j* respectively. *N*_*ij*_ and *M*_*ij*_ are updated as follows:

$$\Delta N_{ij}=\left(1-\rho_{i}\right)\frac{w_{ij}}{\sum_{j}w_{ij}}N_{i}\Delta t-\frac{1}{l_{ij}}N_{ij}\Delta t,$$

$$\Delta M_{ij}=\left(1-\rho_{i}\right)\frac{w_{ij}}{\sum_{j}w_{ij}}M_{i}\Delta t-\frac{1}{l_{ij}}M_{ij}\Delta t.$$

#### Accrual of atrophy.

Tissue loss was modeled as the result of two processes: the direct toxicity from the accumulation of infected agents in region *i* and the deafferentation due to neuronal death in regions connected with region *i*. The incremental atrophy at time *t* over Δ*t* in region *i* is given by the following:

$$\Delta L_{i}\left(t\right)=k_{1}\left(1-e^{-r_{i}\left(t\right)\Delta t}\right)+k_{2}\sum_{j}\frac{w_{ji}}{\sum_{j}w_{ji}}\left(1-e^{-r_{j}\left(t-1\right)\Delta t}\right),$$

where *r*_*i*_(*t*) represents the proportion of misfolded agents in region *i* at time *t*, *k*_1_ is the weight (impact) of aSyn accumulation on tissue loss, and *k*_2_ is the weight (impact) of deafferentation from neighboring regions on tissue loss. Both *k*_1_ and *k*_2_ were set to 0.5 such that accumulation of infected agents and deafferentation had equal effect on the growth of the atrophy simulated by the model.

### Statistical Analyses

#### Demographics and clinical variables.

The demographics and clinical variables were compared between PD patients and controls at baseline and between PD patients at each follow-up timepoint versus baseline. Student’s two-sample *t* tests and Mann-Whitney U tests were respectively used for normally and non-normally distributed continuous variables. Chi-square tests were used for comparing groups on categorical variables.

#### Longitudinal progression of atrophy.

To examine the progression of brain atrophy in PD patients, we performed linear mixed-effect modeling to investigate whether the effect of time was significant over the regional deformation values at each timepoint, namely at baseline and after 1, 2, and 4 years of follow-up. This resulted in a set of 42 separate models, one for each brain region. A random intercept was assigned to each patient, while the fixed effect was the interaction of time with the age-and-sex corrected W-scored DBM values. The Benjamini-Hochberg procedure was used to control the false discovery rate (Benjamini, Drai, Elmer, Kafkafi, & Golani, 2001), and a regional deformation change was considered significant when the *p* value was below 0.05.

#### Fit between observed and modeled pathology.

The SIR model was run for a total of 10,000 iterations after injecting pathology into the substantia nigra. The propagation speed, *v*, which models the protein spreading rate, was set to 1. Variation in propagation speed values ranging from 0.1 to 10 resulted in negligible difference in model fit (Supplementary Figure 1 in the Supporting Information). The model fit between simulated atrophy and observed atrophy progression was measured using Spearman’s rank coefficient correlations by correlating the simulated atrophy at each of 10,000 timesteps to the atrophy progression between baseline and each follow-up timepoint. The peak fit between simulated and atrophy progression patterns corresponded to the highest correlation coefficient between the two metrics.

#### Null models.

To investigate the impact of gene expression and connectivity on the spread of pathologic aSyn, we generated null models in which gene expression or connectivity were randomized and compared the spatial patterns thus obtained to the true peak fits between true and simulated atrophy (Markello & Misic, 2021). For the connectome null models, the impact of topology and/or geometry was investigated using both rewired and repositioned null models. In rewired null networks, using the Maslov-Sneppen algorithm in the Brain Connectivity Toolbox (https://sites.google.com/site/bctnet), pairs of connectivity strength between brain regions were randomly shuffled in the structural connectivity matrix while preserving the network’s original degree sequence and density; the rewiring per edge parameter was set to 100. In repositioned null networks, the spatial position of regions was randomly shuffled while preserving the network’s original degree sequence and connection profile. For gene expression null models, the values of either *SNCA* or *GBA* regional expression were randomly reassigned to each region. For these null models, we additionally used randomly shuffled gene expression values that preserved spatial autocorrelation between regions. This was done by shuffling the gene expression in the BrainSMASH toolbox (Burt, Helmer, Shinn, Anticevic, & Murray, 2020) using the distance matrix used in the SIR model. In every case, the shuffled connectivity of gene expression data was inserted back into the model and used to simulate the spread of agents. For each null model (i.e., rewired, repositioned, *SNCA*, and *GBA* null models), the randomization was repeated 500 times to generate distributions of null peak fits. The original peak fit between the observed and simulated atrophy patterns was then compared using one-sample *t* tests to the average peak fit distributions of the null models.

## RESULTS

### Participants

A total of 1,068 T1-weighted scans from 790 PD patients and 278 controls were obtained from the PPMI cohort (Figure 1). Of these, 199 scans were rejected: 193 failed quality control, and 6 scans were acquired outside the follow-up timepoints investigated in this study (i.e., they were acquired 3 and 5 years after baseline). This yielded a total of 869 scans from 631 PD patients and 238 controls. For the PD group, there were 318 scans at baseline, 120 scans at one year, 108 scans at 2 years, and 85 scans at 4 years. Only patients with a scan at baseline and at one follow-up timepoint were kept for further analysis, leaving samples of 113 patients between baseline and one year, 104 patients between baseline and 2 years, and 79 patients between baseline and 4 years. The complete sample of 157 healthy control participants at baseline was used as the control dataset due to the small number of scans at the follow-up timepoints.

At baseline, there were no significant differences in age, sex, and education between PD patients and controls (Table 1). However, PD patients had significantly higher scores on the MDS-UPDRS-I, MDS-UPDRS-II, MDS-UPDRS-III, Geriatric Depression Scale, and Scales for Outcomes in Parkinson’s Disease-Autonomic. There was also a significantly higher percentage of probable REM sleep behavior disorder in PD patients, as well as lower scores on the Montreal Cognitive Assessment, Symbol-Digit Modalities Test, and the total recall, delayed recall, and recognition tasks from the Hopkins Verbal Learning Test-Revised. In PD patients, scores gradually worsened at each follow-up timepoint on the MDS-UPDRS-I, MDS-UPDRS-II, MDS-UPDRS-III, and the Scales for Outcomes in Parkinson’s Disease-Autonomic.

**Table 1.** Demographics and clinical characteristics of patients and controls. Data are shown as mean (standard deviation). The performance in PD patients at the follow-up timepoint was statistically compared with their performance at baseline. ^a^unpaired *t* test, ^a*^paired *t* test, ^b^chi-square test, ^c^Mann-Whitney U test. BJLO = Benton Judgment of Line Orientation; GDS = Geriatric Depression Scale; HC = healthy controls; HVLT-R = Hopkins Verbal Learning Test-Revised; LNS = Letter-Number Sequencing; MDS-UPDRS = Movement Disorders Society-Unified Parkinson’s Disease Rating Scale; MoCA = Montreal Cognitive Assessment; PD = Parkinson’s disease; RBD = REM sleep behavior disorder; SCOPA-AUT = Scales for Outcomes in Parkinson’s Disease-Autonomic; SDMT = Symbol-Digit Modalities Test; STAI = State-Trait Anxiety Inventory.

**Variables**	**Baseline**			**1-year follow-up**		**2-year follow-up**		**4-year follow-up**	
	**PD (*n* = 318)**	**HC (*n* = 157)**	***p***	**PD (*n* = 113)**	***p***	**PD (*n* = 104)**	***p***	**PD (*n* = 79)**	***p***
Age	60.9 (10.0)	60.1 (11.9)	0.41^**a**^	60.7 (9.5)	<0.001^a*^	62.4 (9.4)	<0.001^a*^	64.6 (9.8)	<0.001^a*^
Sex (% male)	201 (63%)	103 (66%)	0.68^**b**^	72 (64%)		65 (63%)		54 (68%)	
Education, years	15.77 (2.94)	16.06 (2.94)	0.31^**a**^	15.4 (2.8)		15.2 (2.6)		15.5 (2.9)	
MDS-UPDRS-III	18.52 (7.82)	1.14 (2.19)	**<0.001^a^**	21.5 (10.0)	0.001^a*^	23.3 (11.4)	<0.001^a*^	23.4 (10.4)	<0.001^a*^
MDS-UPDRS-II	5.20 (4.06)	0.41 (0.97)	**<0.001^c^**	7.1 (4.6)	**<0.001^c^**	7.3 (4.8)	**<0.001^c^**	9.3 (5.7)	**<0.001^c^**
MDS-UPDRS-I	3.51 (2.70)	2.44 (2.64)	**<0.001^c^**	4.8 (3.2)	**<0.001^c^**	5.0 (3.1)	**<0.001^c^**	6.4 (4.0)	**<0.001^c^**
GDS	2.27 (2.40)	1.13 (2.24)	**<0.001^c^**	2.5 (2.7)	0.43^**c**^	2.4 (2.7)	0.93^**c**^	2.2 (2.1)	0.72^**c**^
STAI	93.52 (7.90)	94.31 (7.14)	0.29^**a**^	92.2 (7.2)	0.24^a*^	92.0 (7.2)	0.14^a*^	92.5 (7.7)	0.31^a*^
SCOPA-AUT	9.27 (5.94)	3.78 (3.92)	**<0.001^c^**	10.3 (5.6)	**<0.001^c^**	10.8 (5.4)	**<0.001^c^**	12.3 (6.2)	**<0.001^c^**
Probable RBD (% cases)	120 (38%)	33 (21%)	**<0.001^b^**	32 (28%)		38 (37%)		34 (43%)	
MoCA	27.4 (2.1)	28.3 (1.1)	**<0.001^c^**	27.1 (2.6)	0.27^**c**^	27.1 (2.3)	0.16^**c**^	27.5 (2.6)	0.64^**c**^
SDMT	41.52 (9.34)	46.9 (11.1)	**<0.001^a^**	41.7 (10.2)	0.67^a*^	41.0 (10.1)	0.11^a*^	40.1 (11.3)	0.02^a*^
LNS	10.73 (2.72)	10.93 (2.64)	0.46^**a**^	10.8 (2.5)	0.08^a*^	10.8 (2.8)	0.37^a*^	10.6 (3.3)	0.12^a*^
BJLO	25.68 (4.18)	26.36 (3.75)	0.06^**c**^	25.2 (4.3)	**<0.01^c^**	25.7 (4.1)	0.19^**c**^	26.2 (3.6)	0.75^**c**^
Semantic fluency	14.52 (4.59)	14.96 (4.15)	0.12^**c**^	14.3 (4.0)	0.85^**c**^	14.7 (4.1)	0.66^**c**^	14.3 (4.6)	0.84^**c**^
Phonemic fluency	13.27 (4.73)	14.04 (4.45)	0.09^**a**^	13.8 (4.4)	0.02^a*^	13.9 (4.6)	<0.01^a*^	14.7 (4.6)	<0.001^a*^
HVLT-R, total recall	24.8 (5.0)	26.0 (4.5)	**0.01^a^**	24.6 (5.4)	0.28^a*^	24.5 (5.7)	0.04^a*^	24.8 (6.0)	0.05^a*^
HVLT-R, delayed recall	8.57 (2.48)	9.27 (2.26)	**0.002^c^**	8.6 (2.6)	0.91^**c**^	8.5 (2.5)	0.76^**c**^	8.5 (3.2)	0.25^**c**^
HVLT-R, recognition	11.24 (1.19)	11.51 (0.82)	**0.006^c^**	11.3 (1.4)	0.27^**c**^	11.3 (1.7)	**0.03^c^**	11.3 (0.9)	0.25^**c**^

### Brain Atrophy Progresses Over 4 Years in PD

**Figure 2.**
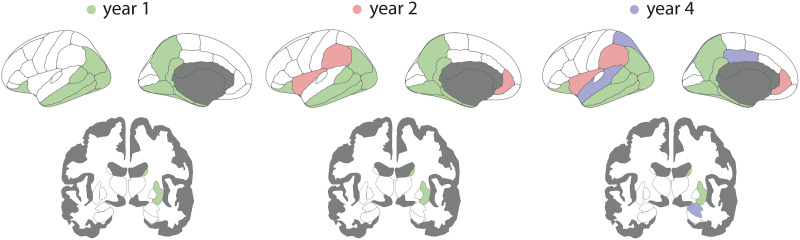
Regional atrophy progression in PD over 4 years. Brain deformation maps showing the regions with significant atrophy progression at each timepoint compared with baseline. Only the left hemisphere is shown. Color reflects the first occurrence of a significant effect. PD = Parkinson’s disease.

Using linear mixed-effect models, 23 of the 42 left hemisphere brain regions showed significant progression of deformation in PD over 4 years, while controlling for age and sex (Figure 2, and Supplementary Table 2 in the Supporting Information). Specifically, between baseline and year 1, atrophy progressed in 14 regions, including the striatum, the temporal areas (i.e., middle and inferior temporal cortices, entorhinal cortex, parahippocampal gyrus, banks of the superior temporal sulcus, lingual, and fusiform gyri), the isthmus of the cingulate cortex, the precuneus and inferior parietal cortex, the lateral occipital cortex, and the lateral orbitofrontal cortex. After 2 years of follow-up, the rostral anterior cingulate cortex, supramarginal cortex, temporal pole, and insula also started showing significant deformation. After 4 years of follow-up, atrophy was now additionally present in the posterior cingulate cortex, superior parietal cortex, superior temporal cortex, nucleus accumbens, and amygdala. Unlike the other regions, the insula demonstrated tissue expansion both at baseline and increasing with time. This tissue expansion may represent an increase in cerebrospinal fluid volume in the perisylvian area. These results confirm previous analyses of this dataset using slightly different methodology (Tremblay et al., 2021).

### The Agent-Based SIR Model Recreates Atrophy Progression

**Figure 3.**
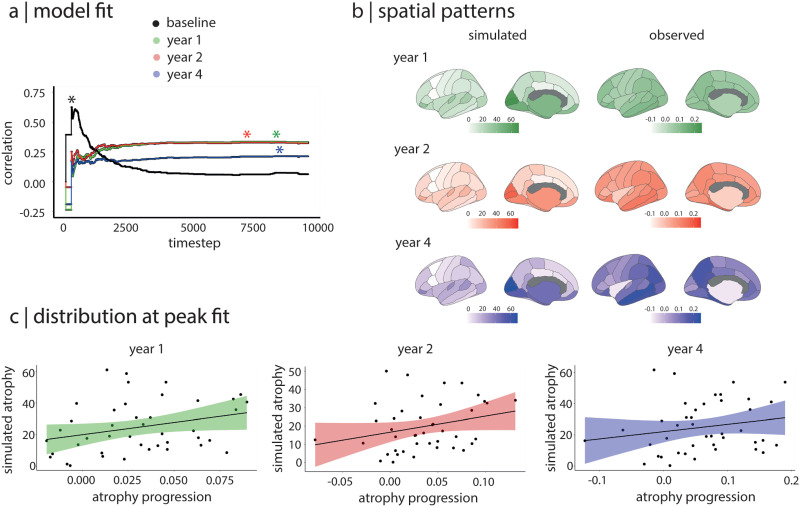
The SIR model recreates the progression of atrophy in PD. (A) Distributions of the correlations between simulated atrophy and observed atrophy progression over the 10,000 simulation timesteps. The asterisks indicate the peak correlation between atrophy patterns; the y-axis represents the Spearman correlation coefficient. (B) Brain plots showing the patterns of simulated atrophy at the peak fit and the patterns of observed atrophy progression for each follow-up timepoint. (C) Relationships between simulated atrophy at the peak fit and observed atrophy progression for each follow-up timepoint. PD = Parkinson’s disease.

Next, we used the agent-based SIR model to simulate the spread of aSyn in the 42 regions and compared the pattern of atrophy simulated by the model with the patterns of atrophy progression observed in PD patients between baseline and 1 year, 2 years, and 4 years of follow-up. We found that the atrophy pattern simulated by the model significantly recreated the atrophy progression patterns observed longitudinally in PD (Figure 3). Specifically, the peak correlation between the simulated atrophy pattern and the progression of atrophy in PD was *r* = 0.34 (*p* = 0.03) at 1 year (Y1) and *r* = 0.33 (*p* = 0.03) at 2 years (Y2, Figure 2B); in contrast to the atrophy seen at baseline, whose peak fit occurred at timestep 228, the peak correlation fits for the 1- and 2-year atrophy progression were reached at much later timesteps (i.e., between timesteps 7,000 and 9,000), once the system has reached its equilibrium state. The simulated atrophy generated by the model did not recreate the pattern of atrophy progression seen between baseline and 4 years (*r* = 0.21, *p* = 0.18). To test the robustness of these results, we repeated the same analyses using structural connectivity matrices containing 20%, 30%, and 40% of the most frequently occurring edges instead of the 35% density used for the main findings. Results were similar (Supplementary Table 1 in the Supporting Information). Taken together, this demonstrates that the agent-based SIR model recreates the progression of brain atrophy taking place over 1 and 2 years in PD.

### Brain Connectivity Shapes the Progression of Atrophy

**Figure 4.**
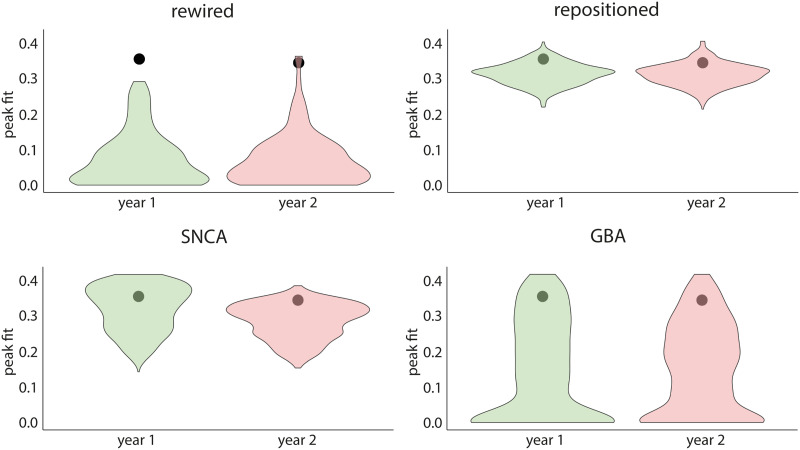
Structural connectivity and gene expression shape atrophy progression in PD. Distributions of null peak correlation fits between simulated atrophy and observed atrophy progression when randomly shuffling the connectivity between regions (rewired null models), the spatial embedding of regions (repositioned null models), and the local expression of *SNCA* or *GBA*. The black circle refers to the true correlation between simulated atrophy and observed atrophy progression. PD = Parkinson’s disease.

To investigate whether the connectome’s architecture shaped the progression of atrophy in PD, we generated sets of 500 rewired and 500 repositioned null models in which the connectome’s topology or geometry was randomized. For the rewired models, the peak fits were always significantly lower than the peak fit obtained with the true connectivity matrix (*p* < 0.0001 for every follow-up timepoint; Figure 4). Using repositioned models to randomize the physical position of brain regions, we also observed that the peak fit was significantly disrupted at every timepoint (*p* < 0.0001; Figure 4). Similar results were found when using other network densities (Supplementary Figure 1 in the Supporting Information). Taken together, this demonstrates that both the brain’s structural connectivity pattern and the physical properties of the connectome contribute to shaping the progression of brain atrophy in PD.

### Gene Expression Shapes the Progression of Atrophy

To investigate whether regional gene expression shaped the progression of atrophy in PD, the expression of *SNCA* or *GBA* was randomized across brain regions while accounting for spatial autocorrelation. The fit between the simulated and observed patterns of atrophy progression was significantly disrupted at baseline and at 1 and 2 years when randomizing *SNCA* (*p* < 0.0001) or *GBA* (*p* < 0.0001) (Figure 4). This suggests that local synthesis and clearance of aSyn, as indexed by regional *SNCA* and *GBA* expression, also contribute to shaping atrophy in PD.

## DISCUSSION

The prion-like model of PD makes three predictions: (a) misfolded aSyn isoforms act as a template to misfold normal aSyn molecules; (b) abnormal aSyn molecules propagate trans-synaptically via the connectome; and (c) accumulation of misfolded aSyn leads to progressive tissue damage in vulnerable regions. We previously developed an agent-based SIR model that simulates the prion-like propagation of aSyn pathology in the brain and recreates atrophy patterns seen at baseline in PD and isolated REM sleep behavior disorder (Rahayel, Tremblay, et al., 2022; Zheng et al., 2019). However, it remains unknown whether the progression of atrophy in PD also follows the constraints imposed by a prion-like spread. In this study, we simulated the propagation of alpha-synuclein pathology in the brain and compared the resulting patterns with maps of atrophy progression over 1, 2 and 4 years in patients with PD.

Three main observations were made: first, atrophy increased significantly over 4 years, being found in the striatum early on and involving a greater number of cortical regions as disease progresses. Second, the SIR model recreated in silico the pattern of atrophy observed longitudinally in PD patients. Third, the SIR model demonstrated that both cell-autonomous factors, here *SNCA* and *GBA* gene expression levels, and non-cell-autonomous factors, namely the topology and geometry of the connectome, both shape the spatiotemporal progression of atrophy in PD. These findings further support the theory of PD as a propagating synucleinopathy.

Using age- and sex-corrected measures of brain deformation, we found that 55% of brain regions showed significant atrophy in PD at some point over the 4-year follow-up. The regions with the strongest progression of atrophy over 4 years were the putamen and caudate and the middle and inferior temporal cortices. Atrophy in cortical regions such as the rostral anterior cingulate cortex and supramarginal cortex appeared after 2 years, whereas the limbic structures (i.e., amygdala and nucleus accumbens) and the superior parietal, posterior cingulate, and superior temporal cortices started showing atrophy after 4 years of follow-up. This atrophy pattern involving basal ganglia first, followed by mostly posterior cortical regions, was also described in a large cohort from the ENIGMA consortium (Laansma et al., 2021), which included PD patients with overall more advanced disease than our de novo cohort. Note that ENIGMA included the PPMI MRI scans used here, although they accounted for only 15% of the 2,357 PD patients included. Interestingly, the substantia nigra, in which signal changes have been associated with parkinsonism in PD (Gaurav et al., 2021), was atrophied at baseline but did not show any atrophy progression during the follow-up years, suggesting that this region may have already reached maximum tissue loss at the time of clinical diagnosis, at least in terms of detectable volume deformation.

PD is pathologically characterized by the accumulation of misfolded aSyn in Lewy bodies and Lewy neurites (Spillantini et al., 1997). Two theories currently exist to explain the aSyn-related pathogenesis in the brain: the prion-like protein propagation and the selective vulnerability hypotheses (Brundin & Melki, 2017; Surmeier et al., 2017). According to the prion-like hypothesis, pathologic aSyn imposes its misfolded conformation onto native proteins that can then spread trans-synaptically, a hypothesis that is supported by several studies in animals (Luk et al., 2012; Masuda-Suzukake et al., 2013; Rahayel, Misic, et al., 2022). More recently, MRI studies performed in humans have shown that the pattern of atrophy observed in de novo patients with PD overlaps with known structural and functional networks (Pandya et al., 2019; Tremblay et al., 2021; Zeighami et al., 2015), suggesting that brain connectivity is a critical determinant of atrophy in synucleinopathies. However, there is also evidence that connectivity alone does not completely explain the pattern of Lewy-related pathology (Henrich et al., 2020), and that intrinsic factors may govern the selective vulnerability of certain regions or cell types (Fu, Hardy, & Duff, 2018; Gonzalez-Rodriguez et al., 2020). While several factors relating to cellular energetics and neurotransmitter metabolism have been proposed (Giguere, Burke Nanni, & Trudeau, 2018), the concentration of normal aSyn and the expression of *SNCA* are also markers of cell vulnerability (Luna et al., 2018).

Our model explicitly incorporates normal aSyn production and breakdown, and randomizing these values degrades its ability to replicate observed atrophy, as does randomizing connectivity values. Applied to de novo PD patients, the SIR model has previously demonstrated that *SNCA* and *GBA* expression and brain connectivity both significantly influence the distribution of atrophy in the brain of patients with PD or isolated REM sleep behavior disorder (Rahayel, Tremblay, et al., 2022; Zheng et al., 2019). Furthermore, the same model was recently used to predict the spread of pathologic aSyn injected in different brain regions of wild-type mice (Rahayel, Misic, et al., 2022). However, no study had yet applied the agent-based model to the analysis of atrophy progression in PD.

We found that the increase in brain atrophy observed at 1 and 2 years was significantly recreated by the model. The use of null networks in which either gene expression or brain connectivity were randomized shows that the atrophy depends upon both connectivity and gene expression. This is in line with similar studies showing that pathology and atrophy occur along brain networks in other neurodegenerative diseases such as frontotemporal dementia (Brown et al., 2019; Shafiei et al., 2021; Zhou, Gennatas, Kramer, Miller, & Seeley, 2012), Alzheimer’s disease (Raj et al., 2015; Vogel et al., 2020; Vogel et al., 2021), and amyotrophic lateral sclerosis (Meier et al., 2020). In contrast to other computational models, which generally simulate the spread of abnormal proteins by relying on a connectivity-based diffusion mechanism, our agent-based model generates a pattern of propagation and atrophy that also takes local vulnerability into account by simulating the synthesis and degradation of aSyn as individual agents.

The progression of atrophy occurring after 4 years could not be replicated by the model. This may be due to the lower number of scans acquired at the four-year timepoint (79 versus 104 at 2 years) causing reduced statistical power. Also, attrition bias may be present whereby the group of PD patients still in the study at year 4 had milder disease (Tremblay et al., 2021). Another possibility is that neuron and synapse loss over time modified neuronal connectivity, leading to inaccuracies in modeling later spread of pathology using a healthy connectome. Future studies could integrate measures of ongoing loss of connectivity and integrate this into the SIR model.

Regional aSyn concentration was modulated in the SIR model to assess regional vulnerability to pathology accumulation. Shuffling the expression level of either *SNCA* or *GBA* resulted in significantly disrupted fit between observed and simulated data, suggesting the importance of expression of both genes in shaping the spatial pattern of disease spread longitudinally. In other words, regional variations in synthesis and clearance of aSyn, as indexed by *SNCA* and *GBA* expression, contribute to the PD atrophy progression pattern in our model. This is consistent with the fact that mutations in these genes are risk factors for genetic forms of PD (Gan-Or, Liong, & Alcalay, 2018; Konno, Ross, Puschmann, Dickson, & Wszolek, 2016; Riboldi & Di Fonzo, 2019).

Similarly, the randomization of connectome topology (via rewired null models) and spatial organization (via spatial null models) resulted in disrupted fit between observed and simulated data. While this is consistent with trans-neuronal propagation of a toxic agent, it can also be explained by other forms of connectivity-related co-atrophy. For example, interconnected areas may share local properties that render them similarly vulnerable to neurodegeneration. These could include glucose metabolism, gene expression, neuronal cell count and shape, synaptic spine density, and other cytoarchitectonic features (Fulcher & Fornito, 2016; Richiardi et al., 2015; Scholtens, Schmidt, de Reus, & van den Heuvel, 2014), which may all influence local vulnerability.

This study has some limitations. First, the PD patients recruited as part of the PPMI study are younger and have less cognitive impairment than the more general population of PD patients (Marek et al., 2018). Nonetheless, PPMI represents the largest longitudinal dataset of PD patients with MRI acquisition and clinical assessments. Second, while the agent-based SIR model included an effect of deafferentation on atrophy, it did not account for the effect of cell loss on synthesis and propagation of agents. Indeed, ongoing cell loss due to the spread of agents might be expected to modify the parameters of the model. For example, the substantia nigra is a source of propagating aSyn in our model, having both high *SNCA* expression and widespread connectivity (Zheng et al., 2019); however, severe cell loss in this region could impact the spread of pathology in later stages of PD, something our model does not incorporate. Future studies should aim at better characterizing the impact of ongoing neurodegeneration by generating a PD-specific structural connectome to assess its influence on the spread of aSyn. Third, gene expression was investigated only for *SNCA* and *GBA* because of their known importance in aSyn synthesis and degradation; future studies should perform a more thorough evaluation of the different genes that may impact aSyn spread. Finally, our model does not consider the synergy between aSyn accumulation and autophagy-lysosomal dysfunction or mitochondrial failure (Hou, Watzlawik, Fiesel, & Springer, 2020; Senkevich & Gan-Or, 2020), which may also display regional variance.

In conclusion, we showed that brain atrophy progresses in PD into patterns that could be recreated by the agent-based SIR model, a computational model that generates in silico the propagation of aSyn and brain atrophy using gene expression and connectivity. This computational model may represent a promising tool for better understanding the mechanisms underlying the progression of atrophy in neurodegenerative diseases.

## DATA AVAILABILITY

W-score deformation maps at each year can be found at https://github.com/alaaabdel/Longitudinal_DBM_Data. The SIR model simulator can be accessed at https://github.com/yingqiuz/SIR_simulator. Any further data are available from the corresponding author upon request.

## AUTHOR CONTRIBUTIONS

Alaa Abdelgawad: Conceptualization; Formal analysis; Software; Visualization; Writing – original draft. Shady Rahayel: Conceptualization; Formal analysis; Investigation; Supervision; Visualization; Writing – original draft. Ying-Qiu Zheng: Conceptualization; Formal analysis; Methodology; Software. Christina Tremblay: Software; Supervision; Validation. Andrew Vo: Investigation; Methodology. Bratislav Misic: Conceptualization; Formal analysis; Investigation; Methodology; Supervision; Writing – original draft. Alain Dagher: Conceptualization; Data curation; Formal analysis; Funding acquisition; Investigation; Methodology; Project administration; Resources; Supervision; Writing – original draft; Writing – review & editing.

## FUNDING INFORMATION

Alaa Abdelgawad, Jeanne-Timmins Costello Fellowship, McGill University. Alain Dagher, CIHR Institute of Neurosciences, Mental Health, and Addiction (https://dx.doi.org/10.13039/501100000034). Alain Dagher, Michael J. Fox Foundation for Parkinson’s Research (https://dx.doi.org/10.13039/100000864). Alain Dagher, Alzheimer’s Association (https://dx.doi.org/10.13039/100000957). Alain Dagher, W. Garfield Weston Foundation (https://dx.doi.org/10.13039/501100000243). Alain Dagher, Healthy Brain for Healthy Lives Initiative (McGill University). Shady Rahayel, Fonds de recherche du Québec–Société et Culture (https://dx.doi.org/10.13039/100008240). Shady Rahayel, Fonds de recherche du Québec–Santé. Shady Rahayel, Canadian Institutes of Health Research.

## TECHNICAL TERMS

Alpha-synuclein: An abundant neuronal protein that may become misfolded and toxic in Parkinson’s disease.
Lewy bodies: Abnormal neuronal inclusions that are a hallmark of Parkinson’s disease neuropathology.
Prions: Misfolded protein isoforms that were discovered as the cause of Creutzfeldt-Jakob disease but may also be implicated in common neurodegenerative diseases.
Susceptible-Infected-Removed (SIR) model: A computational model often used in infectious disease epidemiology.
*SNCA*: The gene for alpha-synuclein.
*GBA*: The gene for glucocerebrosidase, a lysosomal enzyme important for protein degradation.
REM sleep behavior disorder: A condition characterized by moving or talking during sleep, which may be a prodrome of Parkinson’s disease.
Deformation-based morphometry: A method to derive voxel-wise brain atrophy from a single T1-weighted MRI scan.
MDS-UPDRS: A clinical scale used to assess symptom severity in Parkinson’s disease.
Substantia nigra: A nucleus in the midbrain containing dopamine neurons; it is affected early in Parkinson’s disease.
