# Supplementary material for: Predicting longitudinal brain atrophy in Parkinson’s disease using a Susceptible-Infected-Removed agent-based model
Source: Netw Neurosci. 2023 Oct 1;7(3):906–25. doi: 10.1162/netn_a_00296 (PMC10473281; doi:10.1162/netn_a_00296)
Supplement: Supplementary file 1 [file netn-7-3-906-s001.pdf]

Abdelgawad, A., Rahayel, S., Zheng, Y., Tremblay, C., Vo, A., Misic, B. & Dagher, A. (2022). Supporting information for "Predicting longitudinal brain atrophy in Parkinson's disease using a Susceptible-Infected-Removed agent-based model." *Network Neuroscience*. Advance publication. https://doi.org/10.1162/netn_a_00296

Supporting Information

Time point	40% density			35% density			30% density		
	r	timestep	p-value	r	timestep	p-value	r	timestep	p-value
BL	0.58	457	$6.39e^{-5}$	0.58	500	1.24e-5	0.53	260	$3.2e^{-4}$
BL versus Y1	0.31	6417	0.04	0.34	8533	0.03	0.33	9354	0.03
BL versus Y2	0.31	6417	0.04	0.33	7182	0.03	0.33	3252	0.03

Supplementary Table 1. The model recreates atrophy at different network densities. The peak Spearman's correlations between the simulated pattern of atrophy and the patterns of atrophy observed at baseline and at the one- and two-year time points remain when simulating the spread using network densities containing the top 30%, 35%, and 40% connections of the structural connectivity matrix. Timestep refers to the simulation timestep at which peak fit occurs. BL: baseline; Y1: year one; Y2: year two.

	Year 1					Year 2					Year 4				
**Region**	**Coeff**	**SE**	**p**	**95% CI**		**Coeff**	**SE**	**p**	**95% CI**		**Coeff**	**SE**	**p**	**95% CI**	
Lateral orbitofrontal	-0.052	0.023	**0.049**	-0.096	-0.008	-0.073	0.023	**0.006**	-0.119	-0.028	-0.103	0.026	**0.0004**	-0.154	-0.053
Pars orbitalis	-0.035	0.021	0.17	-0.076	0.006	-0.031	0.021	0.24	-0.073	0.011	-0.048	0.024	0.087	-0.094	-0.002
Frontal pole	0.014	0.034	0.76	-0.052	0.081	-0.051	0.035	0.24	-0.12	0.018	-0.039	0.039	0.43	-0.115	0.037
Medial orbitofrontal	-0.019	0.021	0.47	-0.06	0.022	-0.024	0.021	0.37	-0.066	0.018	-0.049	0.024	0.081	-0.095	-0.003
Pars triangularis	0.002	0.017	0.94	-0.031	0.035	0.026	0.017	0.22	-0.008	0.06	0.016	0.019	0.52	-0.022	0.053
Pars opercularis	0.011	0.013	0.52	-0.015	0.037	0.013	0.014	0.44	-0.014	0.04	0.026	0.015	0.17	-0.004	0.055
Rostral middle frontal	-0.037	0.022	0.19	-0.08	0.007	-0.022	0.023	0.44	-0.067	0.023	-0.046	0.025	0.14	-0.095	0.004
Superior frontal	-0.008	0.018	0.76	-0.043	0.027	0	0.018	0.99	-0.036	0.036	-0.028	0.02	0.26	-0.068	0.011
Caudal middle frontal	0.009	0.014	0.63	-0.018	0.036	-0.002	0.014	0.94	-0.029	0.026	-0.009	0.016	0.69	-0.039	0.022
Precentral	0.017	0.016	0.43	-0.016	0.049	-0.002	0.017	0.94	-0.035	0.032	0.004	0.019	0.89	-0.032	0.041
Paracentral	0.001	0.014	0.94	-0.025	0.028	-0.006	0.014	0.76	-0.034	0.22	-0.02	0.016	0.3	-0.051	0.01
Rostral anterior cingulate	-0.03	0.015	0.103	-0.059	0	-0.036	0.016	**0.048**	-0.067	-0.006	-0.075	0.017	**0.00011**	-0.109	-0.042
Caudal anterior cingulate	-0.012	0.01	0.33	-0.032	0.008	0	0.01	0.98	-0.02	0.021	0.006	0.12	0.703	-0.017	0.029
Posterior cingulate	-0.017	0.017	0.44	-0.051	0.017	-0.019	0.018	0.41	-0.054	0.016	-0.054	0.02	**0.017**	-0.093	-0.016
Isthmus of cingulate	-0.057	0.016	**0.001**	-0.088	-0.025	-0.051	0.017	**0.0075**	-0.083	-0.018	-0.119	0.018	**<0.0001**	-0.155	-0.083
Postcentral	0.013	0.016	0.53	-0.019	0.045	-0.009	0.017	0.7	-0.042	0.024	-0.036	0.019	0.109	-0.073	0.001
Supramarginal	-0.035	0.017	0.08	-0.067	-0.002	-0.044	0.017	**0.029**	-0.077	-0.01	-0.077	0.019	**0.00032**	-0.114	-0.04
Superior parietal	-0.2	0.016	0.33	-0.052	0.012	-0.027	0.017	0.19	-0.061	0.006	-0.72	0.019	**0.0007**	-0.109	-0.035
Inferior parietal	-0.049	0.017	**0.01**	-0.082	-0.016	-0.065	0.017	**0.00087**	-0.098	-0.031	-0.142	0.019	**<0.0001**	-0.18	-0.105
Precuneus	-0.047	0.014	**0.002**	-0.074	-0.021	-0.047	0.014	**0.003**	-0.075	-0.02	-0.134	0.015	**<0.0001**	-0.164	-0.103
Cuneus	-0.022	0.013	0.163	-0.083	0.151	-0.013	0.013	0.44	-0.046	0.013	-0.022	0.014	0.202	-0.051	0.006
Pericalcarine	-0.012	0.017	0.59	-0.047	0.022	0.007	0.018	0.79	-0.029	0.042	0.022	0.02	0.39	-0.017	0.061
Lateral occipital	-0.044	0.014	**0.007**	-0.071	-0.016	0.015	0.008	**0.021**	-0.067	-0.01	0.016		**<0.0001**	-0.126	-0.063
Lingual	-0.036	0.015	**0.042**	-0.07	-0.006	-0.03	0.016	0.11	-0.061	0.001	-0.085	0.017	**<0.0001**	-0.119	-0.051
Fusiform	-0.06	0.022	**0.018**	-0.104	-0.017	-0.083	0.023	**0.0013**	-0.13	-0.038	-0.18	0.025	**<0.0001**	-0.229	-0.13
Parahippocampal	-0.077	0.029	**0.02**	-0.13	-0.02	-0.089	0.03	**0.0084**	-0.15	-0.031	-0.16	0.33	**<0.0001**	-0.22	-0.095
Entorhinal	-0.074	0.025	**0.011**	-0.12	-0.024	-0.108	0.026	**0.00032**	-0.16	-0.056	-0.12	0.029	**<0.0001**	-0.18	-0.063
Temporal pole	-0.068	0.03	0.051	-0.126	-0.01	-0.073	0.031	**0.043**	-0.13	-0.013	-0.13	0.034	**0.0007**	-0.2	-0.064
Inferior temporal	-0.074	0.02	**0.001**	-0.113	-0.036	-0.065	0.02	0.0051	-0.105	-0.025	-0.17	0.022	**<0.0001**	-0.21	-0.12
Middle temporal	-0.065	0.017	**0.001**	-0.099	-0.031	-0.081	0.018	**<0.0001**	-0.12	-0.046	-0.18	0.02	**<0.0001**	-0.22	-0.14
Superior temporal sulcus	-0.031	0.012	**0.018**	-0.054	-0.009	-0.039	0.012	**0.0037**	-0.063	-0.016	-0.041	0.013	**0.0057**	-0.067	-0.016
Superior temporal	-0.027	0.017	0.19	-0.06	0.006	-0.026	0.017	0.22	-0.06	0.008	-0.065	0.019	**0.0026**	-0.102	-0.028
Transverse temporal	0.009	0.009	0.44	-0.009	0.027	0.013	0.009	0.29	-0.006	0.031	0.007	0.01	0.64	-0.014	0.027
Insula	0.039	0.027	0.25	-0.041	0.092	0.077	0.028	**0.018**	0.022	0.13	0.116	0.031	**<0.0001**	0.056	0.18
Thalamus	0.01	0.023	0.76	-0.036	0.056	0.009	0.024	0.8	-0.039	0.056	0.06	0.027	**0.057**	0.007	0.11
Caudate	-0.061	0.017	**0.002**	-0.095	-0.027	-0.095	0.018	**<0.0001**	-0.13	-0.06	-0.097	0.02	**<0.0001**	-0.14	-0.058
Putamen	-0.089	0.021	**0.001**	-0.13	-0.048	-0.089	0.022	**0.00032**	-0.13	-0.046	-0.092	0.024	**0.0008**	-0.14	-0.044
Pallidum	-0.004	0.029	0.94	-0.06	0.052	-0.039	0.03	0.29	-0.098	0.019	0.003	0.033	0.95	-0.061	0.067
Accumbens	-0.036	0.029	0.32	-0.093	0.021	-0.066	0.03	**0.06**	-0.13	-0.007	-0.104	0.033	**0.006**	-0.169	-0.039
Hippocampus	-0.038	0.023	0.18	-0.084	0.008	-0.004	0.024	0.93	-0.052	0.043	-0.032	0.027	0.34	-0.084	0.02
Amygdala	-0.006	0.034	0.93	-0.072	0.06	-0.057	0.035	0.19	-0.13	0.012	-0.161	0.038	**0.0002**	-0.24	-0.086
Substantia Nigra	0.005	0.35	0.94	-0.064	0.073	0.045	0.036	0.33	-0.026	0.12	-0.024	0.04	0.66	-0.101	0.054

Supplementary Table 2. Progression of regional brain atrophy in PD. Longitudinal atrophy changes measured using linear mixed effect models in regard to baseline. The significance threshold reported here is presented after FDR correction for multiple comparisons. Coeff = coefficient; FDR = false discovery rate; SE = standard error; CI = 95 % confidence interval.

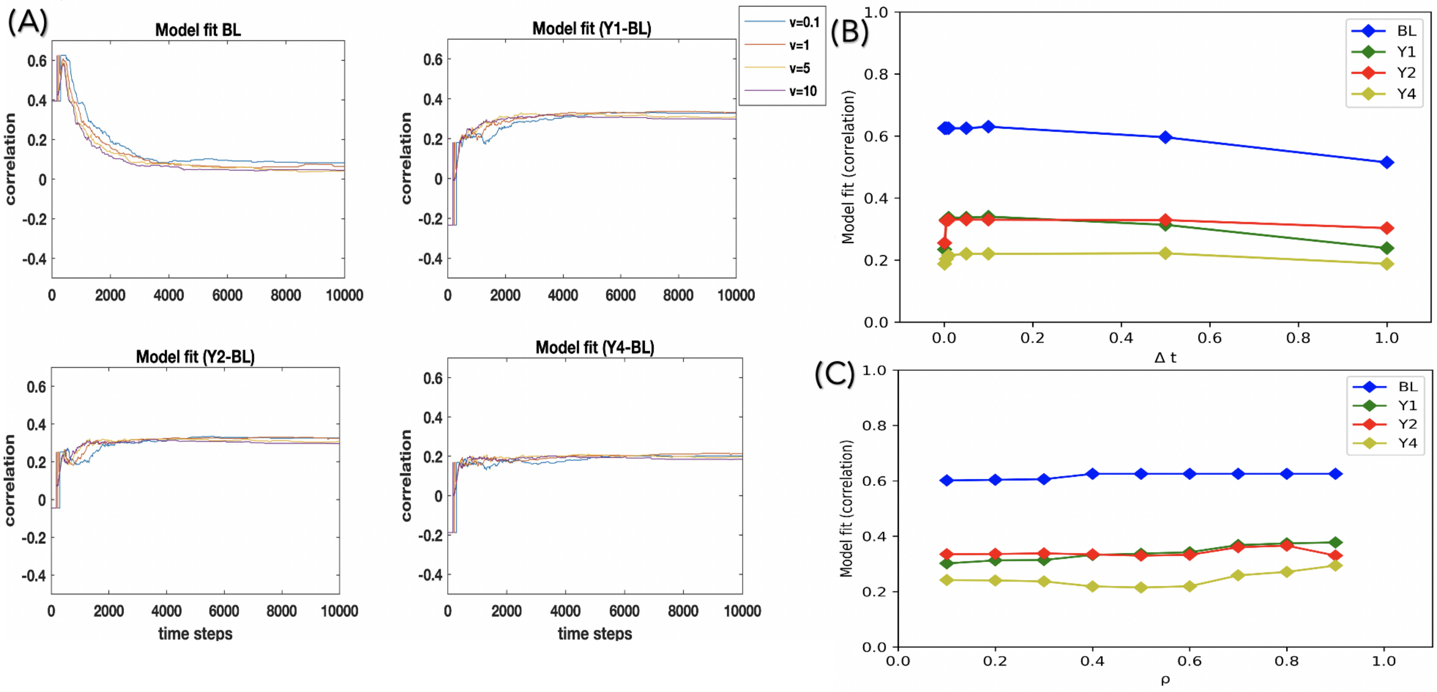

Supplementary Figure 1. Effect of different model parameters. The model fit measured using Spearman's rank correlation coefficients is robust to variations in **(A)** the propagation speed (v), tested using values ranging from 0.1 to 10 (v=1 in the main text), **(B)** the timestep increment (Δt), tested using values ranging from 0.001 to 1 ($\Delta t = 0.01$ in the main text), and **(C)** the probability of an agent staying in region *i* (ρ), tested using values ranging from 0.1 to 0.9 (ρ = 0.5 in the main text) at the connection density of 35% used for main results. All parameters were tested at each time point, with distinct lines indicating peak correlation fits at baseline, at baseline versus one year, at baseline versus two years, and at baseline versus four years of follow-up.
